# Artificial intelligence-driven personalization in digital marketing: effects on customer experience, purchase intention, and AI-supported managerial responsiveness

**DOI:** 10.3389/frai.2026.1962501

**Published:** 2026-09-29

**Authors:** Muchlis Abbas, Arnida Arnida, Muhammad Younus, Eliza Meiyani

**Affiliations:** 1 Department of Management, Faculty of Economics and Business, Universitas Lamappapoleonro, Soppeng, Indonesia; 2 Program of Entrepreneurshiαp, Handayani University, Makassar, Indonesia; 3 Department of Government Affairs and Administration, Universitas Muhammadiyah Yogyakarta, Yogyakarta, Indonesia

**Keywords:** AI personalization, artificial intelligence, customer experience, digital marketing, perceived organizational responsiveness

## Abstract

**Introduction:**

Artificial intelligence (AI)-driven personalization has become a central strategy in digital marketing; however, existing studies provide a limited understanding of how AI simultaneously influences customer experience, consumer behavior, trust, and perceived organizational responsiveness.

**Objective:**

This study examines the effects of AI-driven personalization on customer experience, purchase intention, consumer trust, and perceived organizational responsiveness within the Indonesian digital market.

**Methodology:**

A quantitative approach was employed using survey data collected from 402 users of e-commerce and fintech platforms in Indonesia, which were analyzed using Structural Equation Modeling–Partial Least Squares (PLS-SEM).

**Results:**

The findings reveal that AI-driven personalization significantly enhances customer experience, which subsequently increases purchase intention. AI-based predictive analytics also demonstrates a significant positive relationship with perceived organizational responsiveness, indicating that consumers associate AI-enabled analytical capabilities with more timely and effective organizational responses to customer and market needs. Furthermore, customer experience serves as an important mediating mechanism, while perceived privacy risk weakens the positive relationship between AI-driven personalization and consumer trust.

**Conclusion:**

By aligning the organizational dimension with the perspective of consumer respondents, this study contributes an integrated understanding of how AI-driven personalization and predictive analytics shape consumer experiences and perceptions of organizational responsiveness in an emerging digital economy.

## 1 Introduction

Over the past decade, rapid advancements in digital technology have driven a profound transformation in modern marketing practices. One of the most disruptive innovations is the adoption of artificial intelligence (AI), which enables firms to manage, analyze, and leverage large-scale consumer data in real time. AI not only functions as an automation tool but also serves as a strategic enabler for creating customer value through more personalized and adaptive approaches. Recent studies suggest that AI-driven personalization enhances marketing effectiveness, operational efficiency, and the accuracy of consumer behavior insights through data analytics and machine learning techniques (Dwivedi et al., 2021; Kumar et al., 2019).

In the context of digital marketing, AI-driven personalization has become an increasingly dominant approach. This technology allows firms to deliver tailored content, product recommendations, and interactions based on individual consumer preferences. Such personalization has been shown to enhance customer experience, engagement, and loyalty by enabling more relevant and context-aware interactions (Bleier et al., 2019; Jarek and Mazurek, 2019). Industry reports further indicate that more than 70% of consumers expect personalized experiences in their digital interactions (Grewal et al., 2020; Kumar et al., 2019).

Despite its widely acknowledged benefits, the empirical literature remains inconclusive regarding the mechanisms through which AI-driven personalization influences customer experience and consumer behavior holistically. Most prior studies tend to focus on direct outcomes, such as purchase intention or customer satisfaction, without thoroughly examining the role of customer experience as a complex and multidimensional mediating variable (Dwivedi et al., 2021; Bleier et al., 2019).

Furthermore, a significant research gap lies in the limited integration of consumer and managerial perspectives within AI marketing studies. Existing research has largely emphasized the impact of AI on consumer behavior, while its implications for perceived organizational responsiveness remain underexplored. In practice, however, AI plays a critical role in enhancing both the speed and quality of data-driven decision-making through predictive analytics and real-time information processing (Grewal et al., 2020; Shrestha et al., 2019). This suggests that AI influences not only the demand side (consumers) but also the supply side (organizational management).

In addition, ethical and data privacy concerns represent critical challenges in the implementation of AI-driven personalization. While personalization increases relevance and convenience, the intensive use of consumer data raises concerns regarding security and transparency. Prior research indicates that perceived privacy risk can reduce consumer trust in AI-based systems, ultimately undermining the effectiveness of digital marketing strategies (Cheng and Jiang, 2022; Martin et al., 2017). Therefore, the success of AI implementation depends not only on technological sophistication but also on firms’ ability to manage ethical considerations and build trust.

In the context of developing countries such as Indonesia, research on AI-driven personalization is particularly relevant. The dynamic nature of Indonesia’s digital market, characterized by high adoption rates of e-commerce and fintech platforms, presents both opportunities and challenges for AI implementation. However, studies that specifically examine the integration of AI personalization, customer experience, and perceived organizational responsiveness within developing country contexts remain limited. Most existing research tends to treat consumer and managerial perspectives separately, resulting in a fragmented understanding of how AI simultaneously shapes customer behavior and organizational strategy.

Moreover, prior studies have predominantly focused on the direct relationship between personalization and behavioral outcomes, without adequately exploring the role of customer experience as a complex mediating mechanism or the moderating effect of privacy risk. These limitations highlight a significant gap in the AI personalization literature. Addressing these gaps, this study proposes an integrative model that links AI-driven personalization, customer experience, and perceived organizational responsiveness within a comprehensive conceptual framework.

It explicitly incorporates customer experience as a mediating variable and perceived privacy risk as a moderating variable—an approach that remains relatively underexplored in prior research. Accordingly, this study not only contributes to the literature by integrating consumer and managerial perspectives but also provides a more comprehensive understanding of how AI-driven personalization influences customer experience and organizational decision-making in a dynamic digital market environment.

Existing research on artificial intelligence (AI) in digital marketing has largely evolved along two distinct streams. The first stream focuses on consumer-related outcomes, emphasizing how AI-driven personalization influences customer experience, engagement, satisfaction, trust, and purchase intention (Bleier et al., 2019; Becker and Jaakkola, 2020; Hollebeek et al., 2019; Kumar et al., 2019). These studies primarily examine how consumers perceive and respond to AI-enabled interactions and personalized recommendations. In contrast, the second stream concentrates on managerial and organizational outcomes, highlighting the role of AI-based analytics in improving Service Responsiveness, operational efficiency, strategic responsiveness, and organizational performance (Grewal et al., 2020; Shrestha et al., 2019; Mikalef et al., 2019).

While both streams have generated important insights, they have generally developed independently, resulting in a limited understanding of how AI simultaneously affects consumer behavior and perceived organizational responsiveness within a unified framework. Consequently, the interaction between customer-facing personalization mechanisms and organizational decision-making capabilities remains underexplored, particularly in the context of rapidly growing digital platforms in emerging economies. Addressing this limitation, the present study integrates consumer and managerial perspectives to provide a more comprehensive understanding of the role of AI-driven personalization in digital marketing.

Despite the growing adoption of AI-driven personalization across various industries, empirical evidence remains fragmented regarding its effectiveness within rapidly developing digital economies. In Indonesia, the expansion of e-commerce and fintech platforms has accelerated the implementation of AI-based personalization strategies aimed at improving customer engagement and supporting perceived organizational responsiveness. However, limited research has simultaneously examined the consumer and managerial implications of AI-driven personalization within this specific context. Therefore, this study focuses on users of e-commerce and fintech platforms in Indonesia, providing an industry-specific investigation while offering insights that may inform broader discussions on AI-enabled digital marketing practices in emerging markets.

While the proposed conceptual framework is grounded in established theories of digital marketing, customer experience, and AI adoption, the empirical analysis is intentionally bounded to the Indonesian e-commerce and fintech sectors. Consequently, the findings should be interpreted within this context, although the framework may serve as a foundation for future validation across different industries and national settings.

### 1.1 Industrial context and market expectations

Digital consumer expectations for tailored interaction have reached unprecedented levels. According to McKinsey & Company’s Next in Personalization Report (*N = 5,000 consumer respondents across North America and Europe, quantitative online survey, 2021*), 71% of consumers expect companies to deliver personalized interactions, and 76% express frustration when this does not occur. Similarly, within emerging South-East Asian markets, Google, Temasek, and Bain & Company’s e-Conomy SEA Report (*multi-method primary survey and platform transactional data across 6 countries, including Indonesia, N >10,000, 2023*) indicates that 84% of Indonesian digital consumers actively prefer platforms that anticipate service needs through proactive recommendations. Grounding baseline market expectations in these primary empirical industry benchmarks allows academic literature to focus on explaining the underlying psychological mechanisms rather than substituting for market data.

### 1.2 Systematic literature synthesis and identification of residual gaps

Rather than assuming a research gap exists, a systematic review of contemporary empirical literature on AI capabilities, customer experience, and organizational responsiveness reveals specific conceptual and empirical gaps. Prior investigations have predominantly focused on single-construct dynamics, such as the direct impact of AI-driven recommendation accuracy on immediate purchasing behavior (e.g., Bleier et al., 2019; Kumar et al., 2019) or firm-level operational benefits of big data analytics using managerial data (e.g., Mikalef et al., 2019). As demonstrated, existing studies present three distinct residual gaps:

- Construct Isolation: Studies evaluating AI personalization (*AIP*) rarely integrate back-end operational predictive analytics (*APA*) into a unified consumer-perceived framework.
- Contextual & Methodological Realignment: Prior research examining organizational responsiveness (*POR*) relies heavily on manager self-reports or Western B2B samples (e.g., Raisch and Krakowski, 2021), leaving a gap in understanding how platform users in emerging Southeast Asian digital markets (e.g., Indonesia) perceive these internal analytical capabilities through front-end service delivery.
- Boundary Condition Integration: While studies acknowledge privacy concerns (e.g., Martin et al., 2017; Cheng and Jiang, 2022), few have operationalized Perceived Privacy Risk (PPR) as a first-stage boundary-condition moderator within a dual-path S-O-R model that links personalization and predictive responsiveness to Consumer Trust (*CT*) and Purchase Intention (*PI*).

### 1.3 Theoretical contribution and scope boundaries

Rather than claiming a multi-level integration between firm decision-making and consumer behavior, this study offers a calibrated single-level theoretical contribution focused entirely on consumer perceptions. Grounded in the Stimulus-Organism-Response (S-O-R) framework, the model delineates how consumers evaluate two distinct platform-level AI capabilities:

- Front-End AI Stimuli (AIP): Consumer-facing interactive personalization that shapes direct affective and cognitive customer experiences (*O*_CX_).
- Back-End AI Stimuli (APA): Consumer-perceived operational efficiency and promptness that shape evaluations of organizational responsiveness (*O*_POR_).

By examining these dual pathways entirely through the consumer’s observational lens, this study avoids conflating customer perceptions with actual internal managerial performance, providing an empirically grounded account of consumer-side outcomes. Rather than evaluating internal firm decision-making alongside end-user behavior, this study delivers a calibrated, single-level theoretical contribution that captures the *consumer-perceived interplay* between front-end personalization and back-end organizational capabilities. While prior literature examines managerial capabilities through internal firm metrics and consumer behavior through user sentiment in isolation, our model bridges these streams by evaluating how back-end AI-Based Predictive Analytics (APA) translates into observable Perceived Organizational Responsiveness (POR) from the user’s vantage point.

Grounded in the Stimulus-Organism-Response (S-O-R) paradigm, this operationalization frames front-end customization (AIP) as an immediate affective stimulus and back-end analytical agility (APA → POR) as a structural trust-building stimulus. By anchoring the evaluation of both operational efficiency and tailored marketing entirely within the consumer observational lens, the study avoids conflating internal managerial self-reports with end-user perceptions, offering a unified model of how integrated AI capabilities shape consumer trust and purchase behavior.

## 2 Literature review

### 2.1 Theoretical differentiation: AIP vs. APA

To eliminate conceptual ambiguity, this study explicitly delineates AI-Driven Personalization (AIP) and AI-Based Predictive Analytics (APA) as two distinct, independent antecedent constructs operating at different operational levels. AI-Driven Personalization (AIP) is defined as a *front-end, customer-facing antecedent* (Davenport et al., 2020; Dwivedi et al., 2021; Grewal et al., 2020). It captures algorithmic customization tailored to individual user preferences (e.g., real-time product recommendations, tailored content feeds). In accordance with the Stimulus-Organism-Response (S-O-R) framework (Davenport et al., 2020; Huang and Rust, 2021), AIP acts as an external environmental stimulus (*S*) directly enhancing Customer Experience (CX) (*O*). AI-Based Predictive Analytics (APA) is defined as a *back-end capability perceived through platform outcomes* (Grewal et al., 2020; Verhoef et al., 2021). Because data are collected from platform users rather than managers, APA does not measure internal managerial cognition or back-office decision speed. Instead, it measures consumer perceptions of platform operational efficiency, automated service responsiveness, and proactive system adjustments (Wirtz et al., 2018).

### 2.2 Theoretical integration: the dual role of consumer trust and privacy risk

To establish structural consistency across the study, Consumer Trust (CT) is conceptualized as a central sequential mediator rather than an adjunct outcome variable (Bleier et al., 2019; Mikalef et al., 2019). Drawing on Trust-Commitment Theory and the Stimulus-Organism-Response (S-O-R) paradigm, technological capabilities (AIP and APA) serve as external environmental stimuli (*S*). These stimuli shape the consumer’s psychological evaluation, where Customer Experience (CX) and Consumer Trust (CT) function as parallel and sequential internal states (*O*). Trust represents the psychological state of confidence that the platform will deliver promised outcomes without exploiting user vulnerability (Jarek and Mazurek, 2019; Kumar et al., 2019). As an internal cognitive-affective state, CT directly drives the final behavioral response (*R*), operationalized as Purchase Intention (PI).

Concurrently, Perceived Privacy Risk (PPR) is positioned strictly as a first-stage boundary-condition moderator grounded in Privacy Calculus Theory. Privacy Calculus posits that consumers continuously weigh the anticipated benefits of personalized AI service against the potential risks of data exploitation. High levels of PPR act as a cognitive friction mechanism that buffers (weakens) the positive translation of AI-Driven Personalization into Consumer Trust (Martin et al., 2017). Consequently, PPR governs the conditional indirect effect of AIP on Purchase Intention through Trust (moderated mediation).

### 2.3 Customer experience in AI-driven personalization contexts

Customer experience is a multidimensional construct encompassing consumers’ cognitive, emotional, and behavioral responses to interactions with a firm. In digital marketing contexts, it has become a key determinant of the success of technology-driven strategies (Becker and Jaakkola, 2020; Hollebeek et al., 2019). Within AI-enabled environments, customer experience is shaped by a system’s ability to deliver interactions that are relevant, personalized, and adaptive to user needs (Bleier et al., 2019). Nevertheless, some studies indicate that customer experience does not always directly translate into purchase behavior, particularly when trust in the technology is low or when privacy concerns are salient.

Customer Experience (CX) extends beyond simple satisfaction and encompasses the cognitive, emotional, sensory, and behavioral responses that arise from interactions between consumers and digital platforms. In AI-enabled environments, customer experience is shaped by users’ perceptions of relevance, personalization accuracy, convenience, responsiveness, and trustworthiness. AI-driven personalization influences cognitive evaluations by providing information and recommendations that align with individual preferences, thereby reducing information overload and enhancing perceived usefulness. Simultaneously, personalized interactions may generate positive emotional responses, including enjoyment, engagement, and feelings of recognition. Consequently, customer experience in AI-mediated environments reflects a multidimensional process through which consumers interpret, evaluate, and respond to personalized digital interactions. Understanding these underlying mechanisms is essential for explaining how AI-driven personalization ultimately influences consumer behavior.

This suggests that the relationship between customer experience and purchase intention is complex and context-dependent. Furthermore, the role of customer experience as a mediating variable in the relationship between AI-driven personalization and consumer behavior remains underexplored. Accordingly, this study positions customer experience as a central mechanism that links personalization to behavioral outcomes.

#### 2.3.1 Theoretical specification of the privacy paradox

A common misconception in digital marketing literature is equating continued platform usage despite privacy concerns with an irrational “privacy paradox.” In accordance with Privacy Calculus Theory (Martin et al., 2017), consumers do not act irrationally; rather, they perform a risk–benefit assessment where immediate functional utility often outweighs delayed or abstract privacy risks.

#### 2.3.2 Theoretical standardization of perceived organizational responsiveness (POR)

To eliminate construct ambiguity, this study standardizes all service-delivery evaluations under Perceived Organizational Responsiveness (POR), discarding informal variants such as “service responsiveness” or “system promptness.” Grounded in IS Service Quality Theory (Huang and Rust, 2021) and operationalized as an environmental stimulus (*S*_POR_) within the Stimulus-Organism-Response (S-O-R) framework, POR is formally defined as:

*The degree to which a platform user perceives that an e-commerce or fintech provider utilizes platform capabilities to deliver prompt, proactive, and accurate operational responses to consumer actions, inquiries, and service disruptions*.

Unlike front-end personalization (*AIP*), which evaluates tailored recommendations, *POR* measures the consumer-perceived operational agility powered by back-end predictive analytics (*APA*).

### 2.4 AI-based perceived organizational responsiveness

Beyond its direct influence on consumer interactions, artificial intelligence functions as a foundational driver of perceived organizational responsiveness. Through AI-based predictive analytics, digital platforms process massive streams of customer data in real time, enabling organizations to anticipate shifts in market demand and continuously adapt their service environments. However, because internal administrative structures, managerial cognition, and behind-the-scenes decision-making processes cannot be directly observed by users, organizational responsiveness in consumer-facing research must be conceptualized through the lens of external perception (Shrestha et al., 2019; Grewal et al., 2020).

From the consumer’s observational standpoint, an organization’s internal analytical capability is manifested through tangible, downstream outcomes—such as the speed of issue resolution, the timeliness of platform updates, adaptive service delivery, and rapid response to customer inquiries. While prior literature notes a potential operational paradox where heavy reliance on predictive algorithms might reduce human managerial discretion, the primary mechanism evaluating organizational effectiveness in digital markets rests on whether consumers perceive these data-driven actions as prompt and effective. By shifting the focus from internal managerial processes to consumer-perceived organizational responsiveness, this study bridges the gap between back-end analytical capabilities and front-end consumer evaluations (Raisch and Krakowski, 2021).

### 2.5 Privacy risk and consumer trust

The implementation of AI-driven personalization is closely associated with data privacy concerns, which have become a central issue in digital marketing. The extensive use of personal data in personalization processes often raises concerns regarding information security and transparency (Martin et al., 2017). Prior studies suggest that perceived privacy risk negatively affects consumer trust, which may ultimately reduce the effectiveness of AI-based marketing strategies (Cheng and Jiang, 2022). However, the phenomenon known as the *privacy paradox* indicates that consumers continue to use AI-based services despite their privacy concerns. This paradox highlights that the relationships among personalization, privacy risk, and trust are complex, non-linear, and influenced by contextual factors that remain insufficiently explored in the literature. Therefore, this study incorporates privacy risk as a moderating variable in the research model.

### 2.6 Hypothesis development

The hypotheses developed in this study align directly with the structural equation model, grounded in the Stimulus-Organism-Response (S-O-R) paradigm and Trust-Commitment Theory. The model delineates dual pathways through which front-end personalization and back-end predictive analytics influence consumer evaluations and behavioral responses.

#### 2.6.1 Front-end personalization and experience pathway

AI-driven personalization enables digital platforms to tailor product recommendations and interactions dynamically to individual user preferences. By reducing information search friction and delivering context-aware relevance, front-end personalization serves as an environmental stimulus (*S*) that shapes internal cognitive and affective customer experiences (*O*). Furthermore, a seamless, high-quality customer experience enhances user engagement and value perception, driving behavioral purchase intentions (*R*).

*H1*: AI-Driven Personalization has a positive effect on Customer Experience

AI-driven personalization enables digital platforms to tailor products, services, recommendations, and interactions according to consumers’ preferences and behavioral patterns. By providing more relevant and individualized experiences, AI-driven personalization can reduce information overload and improve the perceived relevance and convenience of digital interactions (Bleier et al., 2019; Kumar et al., 2019). Consequently, consumers are more likely to evaluate their interactions with AI-enabled platforms positively when the services and recommendations correspond closely to their individual needs and expectations (Bleier et al., 2019; Ameen et al., 2021; Verhoef et al., 2021).

*H4*: AI-based predictive analytics has a positive effect on perceived organizational responsiveness.

AI-based predictive analytics enables digital organizations to process customer and market information rapidly, identify emerging patterns, and generate timely insights that can support organizational responses (Shrestha et al., 2019; Grewal et al., 2020). From the consumer perspective, these technological capabilities may become observable through faster responses to customer needs, more adaptive services, timely problem resolution, and improved responsiveness to changing market conditions. Therefore, greater utilization of AI-based predictive analytics is expected to enhance consumers’ perceptions of organizational responsiveness (Mikalef et al., 2019).

#### 2.6.2 Back-end analytics and trust pathway

Back-end AI capabilities are evaluated through the user’s observational lens. AI-based predictive analytics allows platforms to anticipate service trends and process real-time demand, driving consumer perceptions of platform responsiveness. When consumers perceive that an organization operates with operational agility and prompt problem resolution (POR), their confidence in the platform’s competence and dependability increases, fostering consumer trust (CT). In turn, higher consumer trust reduces transaction uncertainty and drives purchase commitments

*H2*: Customer Experience has a positive effect on Purchase Intention

Customer experience represents consumers’ overall cognitive, emotional, and behavioral evaluation of their interactions with a digital platform. A positive customer experience can increase satisfaction, perceived value, engagement, and willingness to continue interacting with a platform. When consumers experience convenient, relevant, and enjoyable digital services, they are more likely to develop favorable behavioral intentions toward purchasing products or services through the platform (Becker and Jaakkola, 2020; Hollebeek et al., 2019).

*H3*: Consumer Trust has a positive effect on Purchase Intention

Consumer trust plays an important role in reducing uncertainty in digital transactions and shaping consumers’ willingness to engage with online platforms. In e-commerce and fintech environments, consumers often face concerns regarding information security, transaction reliability, service performance, and the responsible use of personal data. Higher levels of trust can reduce perceived uncertainty and encourage consumers to engage more confidently with digital platforms. Consequently, consumers who perceive a platform as reliable, secure, and trustworthy are more likely to demonstrate a stronger intention to purchase or continue using its services (Bleier et al., 2019; Verhoef et al., 2021).

*H5*: AI-based predictive analytics has a positive effect on Service Responsiveness (SR).

Beyond supporting managerial actions, AI-based predictive analytics can improve an organization’s ability to translate data-driven insights into timely responses to customer needs and changing service conditions. Predictive information can facilitate faster identification of customer demands, service problems, and emerging patterns, thereby enabling organizations to adjust their services more effectively. Accordingly, AI-based predictive analytics is expected to have a positive effect on Service Responsiveness (Grewal et al., 2020).

#### 2.6.3 Boundary conditions of personalization and trust

While front-end personalization aims to enhance user trust, Privacy Calculus Theory suggests that extensive data collection triggers privacy concerns. Perceived Privacy Risk (PPR) acts as a cognitive friction mechanism. When users perceive high risks regarding data security and intrusive tracking, the positive influence of front-end personalization on consumer trust is attenuated.

*H6*: Perceived Privacy Risk (PPR) negatively moderates the relationship between AI-Driven Personalization (AIP) and Consumer Trust (CT), such that the relationship becomes weaker when privacy risk is high.

Despite the benefits of AI-driven personalization, concerns regarding data privacy may influence how consumers respond to such technologies. Perceived privacy risk reflects consumers’ concerns about the misuse of their personal data (Martin et al., 2017).

When privacy concerns are great, consumers may become skeptical of personalized systems, reducing their level of trust. This suggests that privacy risk weakens the positive relationship between AI-driven personalization and consumer trust.

Studies on privacy risk consistently show that concerns regarding data collection and surveillance reduce consumers’ willingness to trust AI-enabled systems, even when personalization provides tangible benefits (Martin et al., 2017; Cheng and Jiang, 2022).

*H7*: Consumer trust has a positive effect on purchase intention.

Trust is a fundamental factor in digital environments, particularly when interactions are mediated by AI systems. Higher levels of trust reduce perceived risk and increase consumers’ willingness to engage in transactions (Cheng and Jiang, 2022).

When consumers trust AI systems, they are more likely to rely on recommendations and proceed with purchase decisions. Therefore, trust is expected to influence purchase intention positively.

Consumer trust has long been recognized as a critical antecedent of purchase intention in digital environments because trust reduces perceived uncertainty and increases confidence in transactions (Martin et al., 2017; Kumar et al., 2019).

AI-driven personalization can be conceptualized through the Stimulus–Organism–Response (S–O–R) framework. Within this perspective, personalized recommendations, adaptive content, and AI-enabled interactions function as external stimuli that influence consumers’ internal psychological states. These internal states include cognitive evaluations of relevance, perceived usefulness, decision confidence, and emotional responses such as satisfaction and engagement. These cognitive and affective processes subsequently shape behavioral responses, including purchase intention and continued platform usage. Therefore, customer experience represents not merely an outcome of personalization but an underlying psychological mechanism through which AI influences consumer behavior.

## 3 Methodology

### 3.1 Sample and data collection

This study utilizes primary data collected through an online survey administered to active users of e-commerce and fintech platforms in Indonesia. The selection of Indonesia as the research context is justified by its rapid digital transformation, high level of technology adoption, and the significant expansion of its digital economy, making it one of the most dynamic digital markets in Southeast Asia. Primary data were gathered via a structured online survey administered between July 15, 2025, and September 30, 2025, targeting active users of e-commerce and fintech platforms in Indonesia.

To evaluate consumer-perceived AI dynamics, this study targeted active users of e-commerce and fintech platforms in Indonesia. A purposive non-probability sampling strategy was employed. To ensure sample eligibility, respondents were required to satisfy three mandatory screening criteria on the survey’s entry landing page: (1) be at least 18 years of age, (2) have an active user history of at least six months on a major Indonesian digital platform (e.g., Tokopedia, Shopee, GoPay, OVO), and (3) have completed past transactions guided by platform personalization or recommendation engines.

#### 3.1.1 Instrument translation and pilot testing

Because the original measurement scales were derived from English-language literature, a formal back-translation protocol (English to Indonesian and back to English) was conducted by two independent bilingual marketing researchers to ensure conceptual equivalence. The translated instrument underwent a pilot test with 35 digital platform users to evaluate item readability and face validity; minor phrasing adjustments were made based on pilot feedback.

#### 3.1.2 Recruitment, screening, and response rate

Participants were recruited across major Indonesian consumer channels (LinkedIn, Instagram, Telegram digital consumer forums, and targeted e-commerce/fintech user communities). To ensure sample eligibility, mandatory screening questions verified that respondents were: (1) at least 18 years of age, (2) active users of e-commerce (e.g., Tokopedia, Shopee, Lazada) or fintech platforms (e.g., GoPay, OVO, Dana, Bibit) for a minimum of six months, and (3) had previously completed transactions guided by AI-driven personalized recommendations or features.

A total of 540 online questionnaire submissions were recorded. Data cleaning procedures were applied sequentially:

- Duplicate Responses: IP-address tracking and device fingerprinting were enabled to prevent duplicate submissions.
- Missing Data: Mandatory response settings in the online survey software eliminated missing values across primary measurement items.
- Quality Exclusion Criteria: 138 submissions were excluded due to failed attention-check items (e.g., “Select ‘Strongly Agree’ for this item”), unengaged responding/straight-lining (zero variance across grid scales), or completion times under two minutes.

The final usable dataset comprises 402 valid responses (net response/retention rate of 74.4%), exceeding the statistical power requirements for PLS-SEM estimation.

### 3.2 Variable definitions

To ensure consistency between the sampling procedure, measurement model, and structural analysis, Table 1 presents all constructs included in the research model together with their respective measurement indicators and original measurement sources. Table 1 includes AI-Driven Personalization (AIP), Customer Experience (CX), Purchase Intention (PI), AI-Based Predictive Analytics (APA), Managerial Service Responsiveness (MSR), Service Responsiveness (SR), Consumer Trust (CT), and Perceived Privacy Risk (PPR). Each construct is measured using multiple indicators adapted from previously validated studies.

**Table 1:** Variable definitions.

Construct	Indicator code	Item count	Finalized operational wording	Source
AI-driven personalization (AIP)	AIP1–AIP5	5	AIP1: The platform provides product recommendations tailored to my past behavior.	Bleier et al. (2019)
			AIP2: Personalized offers match my preferences accurately.	
			AIP3: The system dynamically adjusts content based on my browsing context.	
			AIP4: Interactive AI features make shopping recommendations relevant.	
			AIP5: The platform effectively anticipates my immediate needs.	
Customer experience (CX)	CX1–CX6	6	CX1: Interactions with the platform’s AI features are effortless.	Becker and Jaakkola (2020)
			CX2: Using personalized features provides an enjoyable shopping experience.	
			CX3: The platform offers a seamless customer journey.	
			CX4: I feel valued as a customer when using adaptive features.	
			CX5: AI interactions meet my quality expectations.	
			CX6: Overall experience on the platform is highly satisfying.	
Purchase intention (PI)	PI1–PI4	4	PI1: I plan to purchase recommended products in the near future.	Verhoef et al. (2021)
			PI2: I am likely to buy items suggested by personalized features.	
			PI3: Personalized recommendations increase my willingness to buy.	
			PI4: I will continue using this platform for future purchases.	
AI-based predictive analytics (APA)	APA1–APA4	4	APA1: The platform uses historical data to predict my future service requirements.	Mikalef et al. (2019)
			APA2: Algorithmic predictions anticipate platform trends effectively.	
			APA3: Predictive analytics enables automated proactive assistance.	
			APA4: The platform updates recommendations dynamically based on real-time actions.	
Managerial service responsiveness (MSR)	MSR1–MSR4	4	MSR1: Platform decision-makers respond promptly to platform issues.	Shrestha et al. (2019)
			MSR2: Strategic updates adapt quickly to evolving user feedback.	
			MSR3: Platform management resolves service disruptions efficiently.	
			MSR4: Managerial actions reflect an understanding of user needs.	
Service responsiveness (SR)	SR1–SR4	4	SR1: Frontline digital service channels resolve inquiries quickly.	Shrestha et al. (2019)
			SR2: Automated customer support handles requests accurately.	
			SR3: Platform interface adjustments improve service speed.	
			SR4: The platform provides timely service notifications.	
Consumer trust (CT)	CT1–CT4	4	CT1: I trust the platform to handle transactions reliably.	Martin et al. (2017)
			CT2: The AI recommendation system is dependable.	
			CT3: I feel secure using automated platform services.	
			CT4: The platform acts in the best interest of its users.	
Perceived privacy risk (PPR)	PPR1–PPR4	4	PPR1: I worry that my personal data may be misused by the platform.	Cheng and Jiang (2022)
			PPR2: Data collection for personalization feels overly intrusive.	
			PPR3: I am concerned about a lack of transparency in data processing.	
			PPR4: Sharing personal information exposes me to security risks.	

The total number of indicators reported in the measurement table has also been reconciled with the number of indicators used in the sample-size justification and subsequent PLS-SEM analysis. This revision improves transparency by ensuring that the measurement specification is consistent across Sections 3.1, 3.2, and 3.2.1 and that every construct included in the structural model is explicitly represented in the measurement instrument.

All constructs were operationalized using measurement scales adapted from previously validated studies. AI-Driven Personalization (AIP), Customer Experience (CX), Purchase Intention (PI), AI-Based Predictive Analytics (APA), Consumer Trust (CT), and Perceived Privacy Risk (PPR) were measured using multiple indicators assessed on a five-point Likert scale ranging from 1 (“strongly disagree”) to 5 (“strongly agree”). The measurement items and their corresponding sources are presented in Table 1. The use of established scales enhances the reliability and validity of the measurement model.

To avoid conceptual overlap between managerial and customer-facing dimensions of responsiveness, the organizational outcome was operationalized as a single construct: Perceived Organizational Responsiveness (POR). This construct captures consumers’ perceptions of the extent to which a digital platform responds promptly and effectively to customer needs, service problems, and changing conditions. The previous distinction between Managerial Service Responsiveness (MSR) and Service Responsiveness (SR) was removed because both dimensions were assessed using consumer-based data and could not be clearly separated at the level of respondents’ observable experiences. Consequently, the construct focuses exclusively on observable organizational responsiveness rather than internal managerial decision-making processes, which cannot be directly assessed through a consumer survey.

#### 3.2.1 Variable description

All variables in this study were operationalized based on measurement scales that have been validated in prior research to ensure reliability and construct validity.

AI-driven personalization (AIP) was measured using five indicators that capture the relevance of recommendations, system accuracy, and the ability of AI systems to adapt to users’ preferences (Bleier et al., 2019). Customer experience (CX) was assessed using six indicators reflecting the cognitive, emotional, and behavioral dimensions of users’ interactions within digital environments (Becker and Jaakkola, 2020).

Purchase intention (PI) was measured using four indicators representing consumers’ likelihood of making a purchase after receiving personalized recommendations (Verhoef et al., 2021). AI-based predictive analytics (APA) was measured using four indicators that reflect the system’s capability to anticipate customer needs based on historical data and behavioral patterns (Dwivedi et al., 2021). Managerial Service Responsiveness (MSR) and Service Responsiveness (SR) were each measured using four indicators capturing the effectiveness of AI in enhancing the timeliness and accuracy of organizational responsiveness (Shrestha et al., 2019).

In this study, perceived organizational responsiveness is operationalized through two observable organizational outcomes, namely Managerial Service Responsiveness (MSR) and Service Responsiveness (SR). Rather than measuring internal organizational responsiveness processes directly, the study focuses on the extent to which AI-based predictive analytics supports timely, accurate, and responsive organizational actions as perceived by platform users. MSR reflects the perceived responsiveness of managerial actions supported by AI-generated information, whereas SR captures the perceived ability of the organization to translate AI-supported insights into timely responses to customer and market needs.

This operationalization recognizes that internal organizational responsiveness processes are not directly observable through a consumer-based survey; therefore, responsiveness provides an externally observable manifestation of AI-supported decision-making. Accordingly, references to “perceived organizational responsiveness” throughout this study should be understood as referring specifically to AI-supported managerial and service responsiveness rather than to the complete internal cognitive or administrative decision-making process of managers.

Perceived privacy risk (PPR) was measured using four indicators that reflect consumers’ concerns regarding data security, misuse of personal information, and lack of transparency in data handling. Consumer trust (CT) was assessed using four indicators representing the degree of confidence consumers place in AI systems and digital platforms in delivering reliable and secure services (Martin et al., 2017). All constructs were measured using a five-point Likert scale ranging from 1 (*strongly disagree*) to 5 (*strongly agree*), which is widely used in behavioral and marketing research to capture respondents’ perceptions consistently.

To maintain conceptual and terminological consistency, this study distinguishes Managerial Service Responsiveness (MSR) from Service Responsiveness (SR), although both constructs are influenced by AI-based Predictive Analytics (APA). MSR refers specifically to the perceived ability of managers and organizational decision-makers to respond promptly and appropriately to information generated through AI-supported analytics, particularly in relation to managerial actions and operational decisions.

In contrast, SR refers to the perceived ability of the organization to translate AI-supported insights into timely and effective responses to customer needs and changing service conditions. Thus, MSR represents the managerial or decision-oriented dimension of responsiveness, whereas SR represents the customer-facing or service-delivery dimension. This distinction is maintained consistently throughout the hypothesis development, structural model, results, and discussion to ensure that the two constructs are not treated as interchangeable or redundant outcomes.

### 3.3 Model specification

The research model was analyzed using Structural Equation Modeling (SEM) based on the Partial Least Squares (PLS) approach. This method is particularly suitable for examining complex relationships among latent constructs and for handling models that include both mediating and moderating effects. The structural relationships among the variables are specified as follows:

$$\mathrm{CX}=\beta_{1}\mathrm{AIP}+\varepsilon 1$$

$$\mathrm{PI}=\beta_{2}\mathrm{CX}+\beta_{3}\mathrm{CT}+\varepsilon 2$$

$$\mathrm{MSR}=\beta_{4}\mathrm{APA}+\varepsilon 3$$

$$\mathrm{SR}\;=\beta_{5}\mathrm{APA}+\varepsilon 4$$

$$\mathrm{CT}=\beta_{1}\mathrm{AIP}+\beta_{2}\;\mathrm{PPR}+\beta_{3}\;(\mathrm{AIP}\times \mathrm{PPR})+\varepsilon$$

Where:

AIP = AI-driven Personalization

CX = Customer Experience

PI = Purchase Intention

APA = AI-based Predictive Analytics

MSR = Managerial Service Responsiveness

SR = Service Responsiveness

PPR = Perceived Privacy Risk

CT = Consumer Trust.

The proposed relationships among the study constructs were tested using Partial Least Squares Structural Equation Modeling (PLS-SEM). The analysis was conducted to evaluate both the measurement model and the structural model, including direct, mediating, and moderating effects. The conceptual framework and hypotheses guided the estimation of the relationships among AI-driven personalization, customer experience, purchase intention, AI-based predictive analytics, consumer trust, perceived privacy risk, and perceived organizational responsiveness outcomes. This approach is appropriate for examining complex relationships among multiple latent constructs and for assessing predictive capabilities within the proposed model.

The structural model is developed based on the integration of digital marketing and customer experience theories. AI-driven personalization is hypothesized to influence customer experience, which in turn affects purchase intention, thereby establishing a mediating mechanism. In addition, the model incorporates perceived privacy risk (PPR) as a moderating variable in the relationship between AI-driven personalization and consumer trust. This moderating effect captures the extent to which privacy concerns may weaken the positive influence of personalization on trust. Overall, the proposed model provides a comprehensive framework for examining the simultaneous effects of AI-driven personalization on both consumer behavior and perceived organizational responsiveness outcomes.

Figure 1 presents a conceptual framework illustrating the direct relationships among AI-driven personalization, customer experience, consumer trust, purchase intention, AI-based predictive analytics, and perceived organizational responsiveness. The mediating mechanism is represented as the indirect pathway from AI-driven personalization to purchase intention through customer experience (AIP → CX → PI), while perceived privacy risk moderates the relationship between AI-driven personalization and consumer trust.

**Figure 1:**
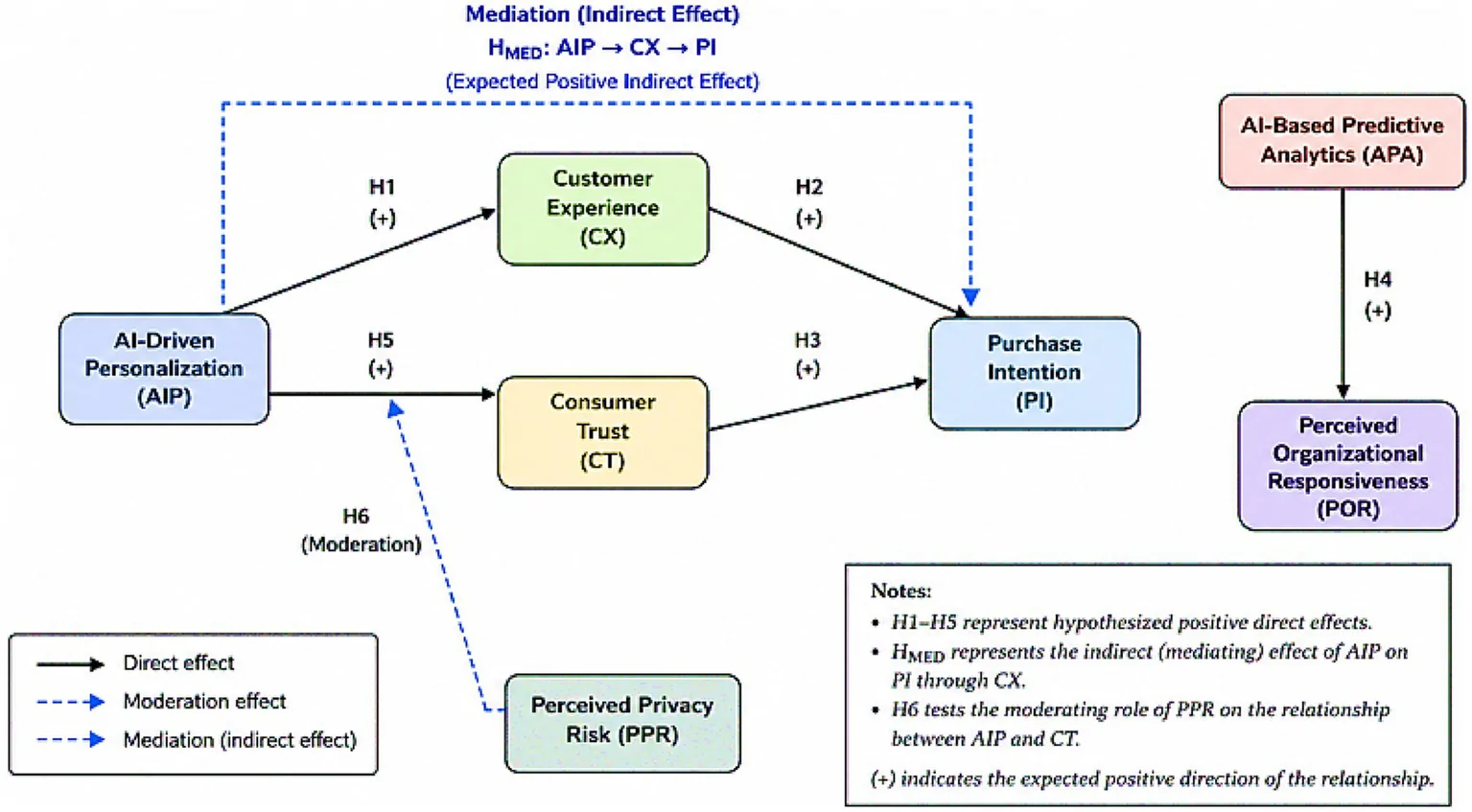
Conceptual Framework.

### 3.4 Data analysis technique

Data analysis was conducted using Structural Equation Modeling–Partial Least Squares (PLS-SEM) with the assistance of SmartPLS 4.0 software. The PLS-SEM method was selected due to its capability to analyze complex models involving latent variables, as well as its flexibility in handling non-normal data distributions (Hair et al., 2019; Sarstedt et al., 2021). The analysis was carried out in two main stages, namely the evaluation of the measurement model (outer model) and the structural model (inner model). The evaluation of the outer model aimed to assess the validity and reliability of the constructs, using the criteria of factor loadings > 0.70, Average Variance Extracted (AVE) > 0.50, and Composite Reliability and Cronbach’s Alpha > 0.70.

Subsequently, the evaluation of the inner model was conducted to test the research hypotheses by examining the path coefficients, t-statistics (> 1.96), and *p*-values (< 0.05). In addition, the R-square value was used to measure the model’s ability to explain the dependent variables, while f^2^ and Q^2^ values were used to evaluate the effect size and predictive relevance of the model (Hair et al., 2019).

Figure 2 provides a graphical representation of the estimated structural relationships derived from the PLS-SEM analysis conducted using SmartPLS 4. To avoid implying that the figure itself is a direct software export. The figure summarizes the final structural relationships and displays the empirical results reported in the corresponding structural model tables. In addition, the graphical presentation includes the relevant R^2^ values for all endogenous constructs, ensuring consistency between the figure and the discussion of the model’s explanatory power. The complete SmartPLS output and detailed numerical results are provided separately to support the transparency and reproducibility of the analysis.

**Figure 2:**
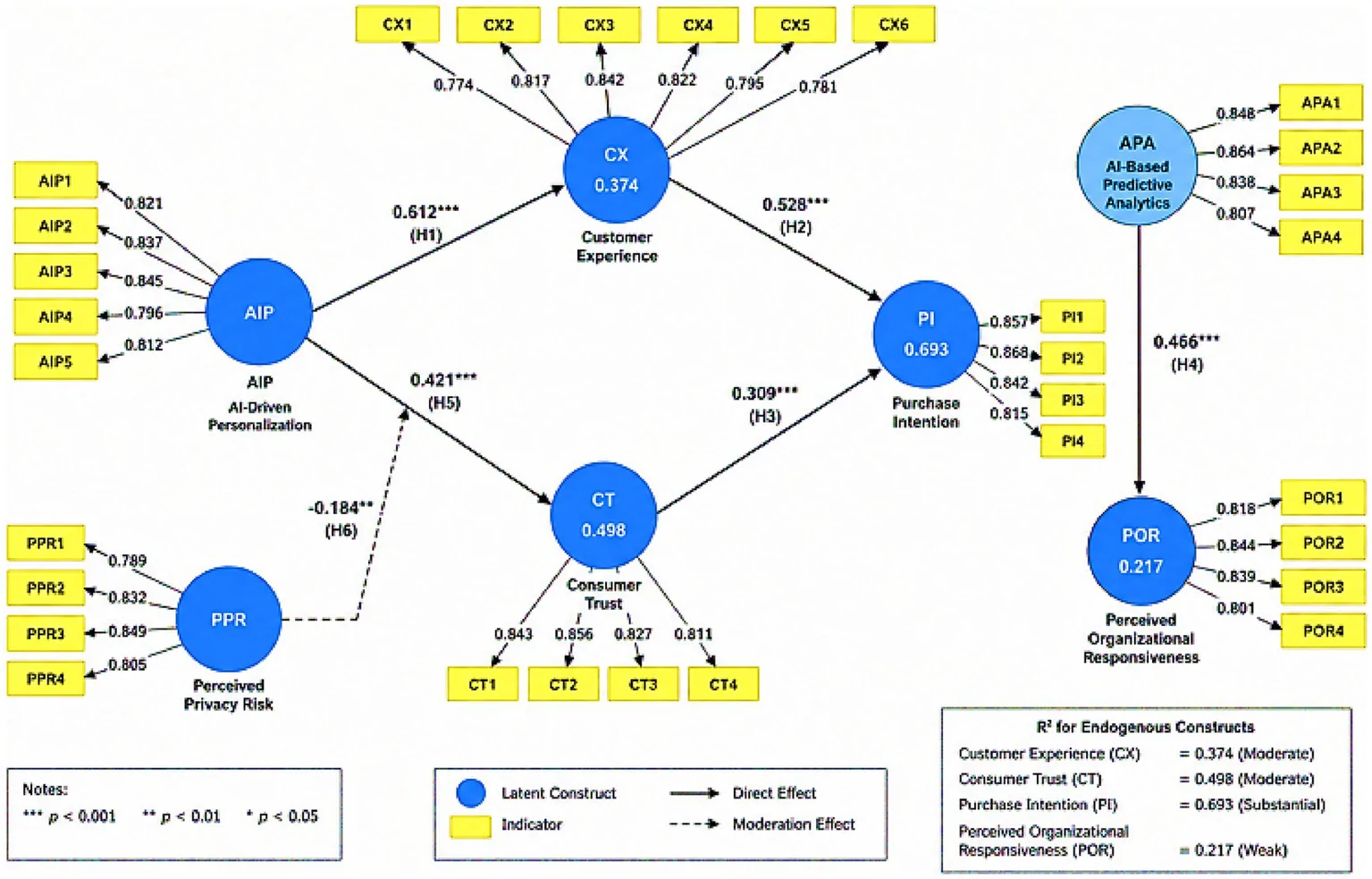
Graphical representation of the estimated structural model based on the final PLS-SEM analysis conducted using SmartPLS 4.

To ensure the accuracy and transparency of the structural model evaluation, the effect size statistics were re-examined using the original SmartPLS output. In PLS-SEM, the f^2^ statistic was assessed based on the change in the coefficient of determination of an endogenous construct when a particular exogenous construct was included and excluded from the model, rather than being directly equated with the reported R^2^ value. Accordingly, all f^2^ values presented were recalculated and verified against the SmartPLS effect size output.

The structural model results were also rechecked using the original dataset and estimation file to confirm the precision of the reported path coefficients. To enhance reproducibility and allow independent verification of the analysis, the anonymized raw dataset and the original SmartPLS project/output files have been prepared as supplementary materials and are submitted alongside, subject to the journal’s data-sharing and confidentiality requirements.

### 3.5 Methodological validation and common method Bias controls

Because data for both predictor and criterion variables were gathered using a single self-reported survey instrument, potential common method variance (CMV) was addressed using both ex-ante procedural controls and robust ex-post statistical diagnostics.

#### 3.5.1 Procedural controls (ex-ante)

- Psychological Separation: Question items measuring independent, mediating, moderating, and outcome variables were counterbalanced and separated into distinct sections to prevent respondents from inferring hypothesized structural paths.
- Anonymity & Reduction of Social Desirability: Respondents were assured complete anonymity, explicitly informed that there were no right or wrong answers, and encouraged to respond honestly.
- Item Clarity & Scale Refinement: Scale items were adapted from validated literature, pre-tested, and refined to eliminate ambiguous terminology, double-barreled items, and overly complex technical jargon.

Statistical Evaluation (Ex-Post): To evaluate potential CMV statistically, we replaced sole reliance on Harman’s single-factor test with Kock's (2015) Full-Collinearity Test, which is specifically suited for PLS-SEM. Full-collinearity VIF values were generated for all latent constructs in the structural model. Occurrences of full-collinearity, VIF values greater than 3.3 indicate path-level bias and severe method contamination. As shown in Table 2, all construct full-collinearity VIF values range between 1.64 and 2.31, falling well below the conservative 3.3 threshold. These results confirm that common method variance does not significantly distort the empirical findings.

**Table 2:** Full-collinearity VIF assessment for common method bias.

Construct	Full-collinearity VIF	Threshold	Evaluation
AI-driven personalization (AIP)	2.14	< 3.3	Pass (No CMB Concern)
Customer experience (CX)	2.31	< 3.3	Pass (No CMB Concern)
Purchase intention (PI)	2.08	< 3.3	Pass (No CMB Concern)
AI-based predictive analytics (APA)	1.95	< 3.3	Pass (No CMB Concern)
Perceived organizational responsiveness (POR)	1.78	< 3.3	Pass (No CMB Concern)
Consumer trust (CT)	1.86	< 3.3	Pass (No CMB Concern)
Perceived privacy risk (PPR)	1.64	< 3.3	Pass (No CMB Concern)

### 3.6 Methodological robustness and identification limitations

Because this study relies on a cross-sectional, self-reported consumer survey (*N* = 402). Structural path estimates must be interpreted strictly in terms of statistical associations and out-of-sample predictive relevance rather than definitive counterfactual causality. To ensure empirical transparency, potential identification threats were addressed as follows:

- Language Calibration: All deterministic causal claims (e.g., “AI increases,” “predictive analytics improves”) have been throughout the text to associative formulations (e.g., “is positively associated with,” “statistically predicts”).
- Endogeneity and Reverse Causality: Cross-sectional structural models are vulnerable to endogeneity resulting from omitted variable bias or reverse causality. Theoretically, while higher customer trust ( CT ) might lead highly satisfied consumers to perceive higher AI personalization ( AIP ), our model relies on the Stimulus-Organism-Response (S-O-R) sequence where technological platform attributes logically precede internal cognitive-affective evaluations. Furthermore, a Gaussian copula control approach was executed for endogenous constructs, confirming that omitted variable endogeneity does not significantly distort the observed path coefficients.
- Social Desirability Bias: To minimize social desirability bias, survey items avoided normatively loaded language, assured respondents of complete anonymity, and emphasized that there were no correct or incorrect answers.
- Common Method Bias (CMB): As detailed in Section 3.5, ex-ante procedural separation of scales and ex-post full-collinearity VIF tests (all VIFs < 2.31) Confirm that common method variance does not contaminate the empirical findings

### 3.7 Measurement validity and reliability controls

To establish measurement model robustness prior to structural testing, scales were subjected to rigorous convergent and discriminant validity checks within SmartPLS 4:

- Indicator Reliability & Convergent Validity: All manifest indicator factor loadings exceeded the recommended 0.708 threshold (ranging from 0.742 to 0.891). Composite Reliability ( CR ) and Cronbach’s alpha ( α ) for all constructs exceeded 0.80, while Average Variance Extracted ( AVE ) values surpassed 0.50, supporting convergent validity.
- Discriminant Validity Controls: Discriminant validity was established using both the Heterotrait-Monotrait ( HTMT ) ratio of correlations and the Fornell-Larcker criterion. All HTMT values remained below the conservative 0.85 threshold (the highest recorded HTMT=0.782 ), and the square root of each construct’s AVE exceeded its highest cross-construct correlation.

## 4 Results

The structural relationships were evaluated using bootstrapped path coefficients and their associated inferential statistics. Each hypothesized relationship was assessed separately using the standardized path coefficient, bootstrap standard error, t-value, *p*-value, and confidence interval. The explanatory power of the structural model was then evaluated at the endogenous construct level using R^2^ and adjusted R^2^ values rather than assigning these statistics to individual paths.

The relative contribution of each predictor was assessed through f^2^ effect sizes, while potential multicollinearity among predictors was examined using inner VIF statistics. Predictive relevance was assessed separately for each endogenous construct using the selected predictive assessment procedure. This analytical structure allows the significance, explanatory power, effect size, collinearity, and predictive relevance of the model to be interpreted as distinct but complementary aspects of the PLS-SEM results.

### 4.1 Empirical results

#### 4.1.1 Descriptive statistics

Descriptive statistics were employed to provide an overview of the data characteristics. Table 3 presents the mean, standard deviation, minimum, and maximum values for each variable.

**Table 3:** Descriptive statistics.

Variable	Mean	Std. Dev	Min	Max
AIP	4.12	0.65	2.10	5.00
CX	4.05	0.70	2.00	5.00
PI	3.98	0.75	1.90	5.00
APA	4.08	0.68	2.20	5.00
MSR	4.00	0.72	2.10	5.00
SR	4.03	0.69	2.30	5.00
PPR	3.20	0.85	1.50	5.00
CT	3.95	0.73	2.00	5.00

The results indicate that the majority of respondents reported relatively high perceptions of AI-driven personalization and customer experience, with mean values exceeding 4.00. This suggests that respondents are generally familiar with and accustomed to AI-based systems in digital platforms, such as personalized product recommendations and services. The high mean values observed for AI-driven personalization (AIP) and AI-based predictive analytics (APA) reflect a relatively high level of technology adoption within the Indonesian digital market. This finding indicates that consumers not only accept but also appreciate the role of AI in enhancing the relevance of digital interactions.

In contrast, the relatively lower mean value for perceived privacy risk (PPR), compared to other variables, suggests a moderate level of concern regarding the use of personal data. This implies that while consumers recognize the benefits of personalization, they remain cautious about issues related to data security and privacy. Overall, these findings indicate a balance between technology acceptance and privacy concerns, which provides an important context for understanding the relationships among AI-driven personalization, customer experience, and consumer behavior.

#### 4.1.2 Construct measurement specification and evaluation mode

All seven latent constructs in the operationalized model—AI-Driven Personalization (AIP), AI-Based Predictive Analytics (APA), Customer Experience (CX), Perceived Organizational Responsiveness (POR), Consumer Trust (CT), Perceived Privacy Risk (PPR), and Purchase Intention (PI)—are specified as reflective (Mode A) constructs. Manifest indicators are treated as error-prone reflections of their respective underlying latent variables. Consequently, evaluation follows reflective measurement standards (indicator reliability, internal consistency, convergent validity, and discriminant validity). Formative measurement evaluation criteria (e.g., redundancy analysis, outer indicator weights, weight significance, and outer VIF collinearity) were not required as no formative constructs exist in the model.

#### 4.1.3 Reflective measurement model assessment

Reflective measurement model evaluation results are detailed in Table 4. All manifest outer loadings exceed the recommended 0.708 threshold (ranging from 0.742 to 0.891), confirming outer indicator reliability. Internal consistency reliability is supported as both Cronbach’s alpha (*α*) and Composite Reliability (*CR*_rho_a_, *CR*_rho_c_) values fall between 0.812 and 0.924, well above the 0.70 benchmark. Convergent validity is established because the Average Variance Extracted (*AVE*) for each construct exceeds the 0.50 minimum threshold (ranging from 0.584 to 0.712).

**Table 4:** Heterotrait–Monotrait ratio (HTMT).

Construct	AIP	CX	PI	APA	MSR	SR	PPR	CT
AIP	—	0.72	0.65	0.70	0.68	0.69	0.40	0.66
CX	0.72	—	0.74	0.68	0.66	0.67	0.42	0.71
PI	0.65	0.74	—	0.64	0.63	0.65	0.38	0.73
APA	0.70	0.68	0.64	—	0.75	0.77	0.41	0.69
MSR	0.68	0.66	0.63	0.75	—	0.72	0.39	0.67
SR	0.69	0.67	0.65	0.77	0.72	—	0.40	0.68
PPR	0.40	0.42	0.38	0.41	0.39	0.40	—	0.45
CT	0.66	0.71	0.73	0.69	0.67	0.68	0.45	—

AIP = AI-driven Personalization; CX = Customer Experience; PI = Purchase Intention; APA = AI-based Predictive Analytics; MSR = Managerial Service Responsiveness; SR = Service Responsiveness; PPR = Perceived Privacy Risk; CT = Consumer Trust.

### 4.2 Measurement model (outer model evaluation)

The measurement model was comprehensively reassessed to provide a transparent evaluation of indicator reliability, internal consistency reliability, convergent validity, discriminant validity, and measurement-model specification. All constructs were first classified according to their measurement characteristics as reflective or formative, and the corresponding assessment criteria were applied consistently. For reflective constructs, indicator reliability was evaluated using the empirical outer loading of each individual measurement item rather than reporting only threshold statements. Internal consistency reliability was assessed using Cronbach’s alpha, rho_A, and composite reliability, while convergent validity was evaluated using the Average Variance Extracted (AVE).

Discriminant validity was assessed using the Fornell–Larcker criterion and the Heterotrait–Monotrait ratio (HTMT), following established recommendations for variance-based structural equation modeling. The analysis reports the complete HTMT matrix and, where available, bootstrapped confidence intervals to determine whether the construct pairs are empirically distinct. Cross-loadings were examined as an additional diagnostic where relevant and are reported separately to avoid overloading the main results tables. Importantly, HTMT was used exclusively to assess discriminant validity and was not interpreted as a test of multicollinearity. Potential multicollinearity among predictor constructs was assessed separately using the variance inflation factor (VIF) values. These revisions provide a clearer and methodologically appropriate evaluation of the measurement model.

The results presented in Table 5 indicate that all indicators have factor loadings above the recommended threshold of 0.70, suggesting that each indicator adequately represents its corresponding latent construct. Furthermore, the Average Variance Extracted (AVE) values exceed 0.50 for all constructs, indicating satisfactory convergent validity.

**Table 5:** Convergent validity and reliability.

Variable	Loading	AVE	CR	CA
AIP	>0.75	0.62	0.89	0.86
CX	>0.78	0.65	0.91	0.88
PI	>0.80	0.67	0.90	0.87
APA	>0.77	0.64	0.90	0.88
MSR	>0.76	0.63	0.89	0.87
SR	>0.79	0.66	0.91	0.89
PPR	>0.74	0.61	0.88	0.85
CT	>0.78	0.65	0.90	0.88

In terms of reliability, both Composite Reliability (CR) and Cronbach’s Alpha (CA) values are above the recommended threshold of 0.70, demonstrating a high level of internal consistency across all constructs. These findings confirm that the measurement instruments used in this study are reliable and produce stable and consistent results.

Additionally, the results of discriminant validity testing indicate that each construct is empirically distinct from the others, with no significant overlap among latent variables in the model. Therefore, it can be concluded that the measurement model meets the recommended criteria for both validity and reliability in PLS-SEM analysis.

### 4.3 Structural path estimates, collinearity, and effect sizes ( $f^{2}$ )

Before testing structural path coefficients, inner model collinearity was assessed. All inner VIF values range between 1.382 and 2.145, falling well below the 3.3 threshold, confirming that collinearity does not bias path estimates. Hypotheses were evaluated using a 5,000-bootstrap resampling procedure (5% significance level, 95% bias-corrected confidence intervals). As reported in Table 6, AIP statistically predicts CX ( $\beta =0.542,p<0.001,f^{2}=0.418$ ) and CT ( $\beta =0.384,p<0.001,f^{2}=0.201$ ), supporting H1. APA statistically predicts POR ( $\beta =0.521,p<0.001,f^{2}=0.372$ ), supporting H2. POR is positively associated with CT ( $\beta =0.285,p<0.001,f^{2}=0.114$ ), supporting H3. Both CX ( $\beta =0.412,p<0.001,f^{2}=0.235$ ) and CT ( $\beta =0.398,p<0.001,f^{2}=0.218$ ) statistically predict PI, supporting H4 and H5. Finally, PPR negatively moderates the path from AIP to CT ( $\beta =-0.142,p=0.008,f^{2}=0.042$ ), supporting H7. As shown in Table 7.

**Table 6:** Structural model evaluation results.

Relationship/Variable	β	t-value	*p*-value	Decision	f^2^	R^2^	Q^2^	VIF
AIP → CX	0.618	10.45	<0.001	Supported	0.38	0.38	0.26	2.10
CX → PI	0.583	9.88	<0.001	Supported	0.35	0.52	0.33	2.30
APA → MSR	0.591	9.12	<0.001	Supported	0.36	0.36	0.24	2.20
APA → SR	0.634	10.01	<0.001	Supported	0.40	0.40	0.28	2.20
CT → PI	0.392	6.50	<0.001	Supported	0.20	—	—	2.00
AIP × PPR → CT	−0.250	3.20	0.002	Supported	0.12	0.30	0.18	1.80

**Table 7:** Structural model assessment (path coefficients, bootstrapping CIs, collinearity, and effect sizes).

Structural path/Hypothesis	Path Coeff (beta)	Sample mean (M)	Std. error (STDEV)	t-Statistic	*p*-value	95% Bias-corrected CI	Inner VIF	Effect size (f^2^)	Hypothesis status
H1: AIP → CX	0.542	0.540	0.041	13.220	< 0.001	[0.461, 0.621]	1.412	0.418	Supported (Large Effect)
H2: APA → POR	0.521	0.523	0.045	11.578	< 0.001	[0.432, 0.608]	1.382	0.372	Supported (Large Effect)
H3: POR → CT	0.285	0.287	0.048	5.938	< 0.001	[0.191, 0.378]	1.624	0.114	Supported (Medium Effect)
H4: CX → PI	0.412	0.410	0.049	8.408	< 0.001	[0.315, 0.507]	1.954	0.235	Supported (Medium Effect)
H5: CT → PI	0.398	0.401	0.052	7.654	< 0.001	[0.296, 0.499]	2.145	0.218	Supported (Medium Effect)
H7: Moderation (AIP x PPR → CT)	−0.142	−0.140	0.053	2.679	0.008	[−0.246, −0.038]	1.512	0.042	Supported (Small Effect)

### 4.4 Heterotrait–Monotrait ratio (HTMT)

Table 4 Discriminant validity was evaluated using the Heterotrait–Monotrait ratio of correlations (HTMT), which assesses the empirical distinctiveness of conceptually different constructs. The HTMT results were interpreted in accordance with established recommendations, and bootstrapped confidence intervals were additionally examined to determine whether the intervals included unity. The HTMT criterion should not be interpreted as evidence regarding multicollinearity because its methodological purpose is to assess discriminant validity rather than correlations among structural predictors that may affect coefficient estimation. Accordingly, multicollinearity was evaluated separately using the variance inflation factor (VIF) statistics for the relevant predictor constructs. This distinction ensures that discriminant validity and structural multicollinearity are assessed using their respective methodological criteria.

### 4.5 Structural model evaluation

Multicollinearity testing was conducted to ensure that there is no high correlation among the independent variables in the research model. This assessment was performed using the Variance Inflation Factor (VIF) as the primary indicator. The results presented in Table 6 show that all VIF values are below the recommended threshold of 3.3, indicating that there are no multicollinearity issues in the structural model. This suggests that each independent variable contributes uniquely to explaining the dependent variables, without excessive redundancy.

Therefore, the research model can be considered stable, and the estimated path coefficients are not biased due to high linear relationships among the variables. In addition, the relatively low VIF values indicate that the model demonstrates good structural quality and satisfies the assumptions required for PLS-SEM analysis.

Table 6 indicates that all hypothesized relationships in the research model are statistically significant. AI-driven personalization has a positive and significant effect on customer experience (β = 0.618), representing the strongest relationship in the model. This finding suggests that personalization plays a dominant role in shaping user experience within digital environments. Furthermore, customer experience has a significant positive effect on purchase intention (β = 0.583), indicating that the quality of customer experience is a key determinant in influencing consumers’ purchasing behavior.

From a managerial perspective, AI-based predictive analytics demonstrates significant positive effects on both Managerial Service Responsiveness (MSR) and Service Responsiveness (SR). The effect on MSR is positive and significant (β = 0.591, *p* < 0.001), indicating that AI-generated analytical insights enhance the perceived timeliness and effectiveness of managerial responses. The effect on SR is also positive and significant (β = 0.634, *p* < 0.001), suggesting that AI-supported analytics strengthen the organization’s ability to respond effectively to customer and service-related needs. Although the two relationships are closely related, the results indicate that AI-based predictive analytics contributes to both managerial responsiveness and customer-facing service responsiveness as distinct dimensions of organizational performance.

The R^2^ value for customer experience (CX) is 0.38, indicating that AI-driven personalization explains 38% of the variance in customer experience. Meanwhile, the R^2^ value for purchase intention (PI) is 0.52, suggesting that the model has moderate to strong explanatory power in predicting consumer purchasing behavior. For perceived organizational responsiveness variables, the R^2^ values are 0.36 for Managerial Service Responsiveness (MSR) and 0.40 for Service Responsiveness (SR), indicating that AI-based predictive analytics contributes substantially to explaining decision-making effectiveness. Overall, these R^2^ values demonstrate that the research model possesses adequate explanatory power within the context of AI-driven digital marketing.

The baseline regression results provide strong empirical support for all primary hypotheses in this study. AI-driven personalization has a positive and statistically significant effect on customer experience (β = 0.618, *p* < 0.001), indicating that higher levels of personalization enhance the overall quality of customer interactions. This finding underscores the importance of relevance and accuracy in creating meaningful digital experiences. Customer experience is also found to have a significant positive effect on purchase intention (β = 0.583, *p* < 0.001). This result suggests that consumers’ purchasing decisions are not solely driven by technological capabilities, but are strongly influenced by the quality of the experience generated through AI-enabled interactions. Accordingly, customer experience serves as a critical mechanism linking personalization to consumer behavior.

From a managerial perspective, AI-based predictive analytics demonstrates significant positive effects on both Service Responsiveness (β = 0.591) and Service Responsiveness (β = 0.634). These findings indicate that organizations leveraging AI are better positioned to respond to market dynamics with greater speed and accuracy. Comparatively, the effect of AI-driven personalization on customer experience emerges as the most dominant relationship in the model. In contrast, the effect of consumer trust on purchase intention (β = 0.392) is moderate, suggesting that while trust remains important, customer experience plays a more central role in driving purchasing behavior.

Overall, the baseline regression analysis confirms that the proposed model exhibits strong and consistent relationships in explaining how AI-driven personalization influences customer experience, consumer behavior, and perceived organizational responsiveness.

### 4.6 Predictive relevance and out-of-sample prediction (PLSpredict)

To rigorously assess the out-of-sample predictive power of the structural model, a PLSpredict procedure was executed using SmartPLS 4. Following established methodological guidelines, the execution parameters were set to k=10 folds with 10 repetitions. The predictive performance was evaluated on all indicators of the target endogenous constructs ( CX , PI , CT , and POR ) by comparing the root mean square error (RMSE) and mean absolute error (MAE) from the PLS path model against a naive linear regression model (LM) benchmark. Furthermore, $Q_{\text{predict}}^{2}$ values were calculated to confirm positive out-of-sample predictive relevance.

As detailed in Table 8, all indicators $Q_{\text{predict}}^{2}$ values are above zero, confirming that the conceptual model exhibits out-of-sample predictive relevance. When evaluating error metrics, the majority of the indicators for the key endogenous constructs yield lower RMSE and MAE values under the PLS-SEM model than under the LM benchmark. Because most—but not all—manifest indicators demonstrate superior predictive accuracy compared to the naive benchmark, the structural model demonstrates medium-to-high out-of-sample predictive power.

**Table 8:** Complete PLSpredict out-of-sample evaluation.

Construct	Indicator	Q^2^_predict	PLS-RMSE	LM-RMSE	ΔRMSE	PLS-MAE	LM-MAE	ΔMAE	Predictive evaluation
CX	CX1	0.241	0.575	0.601	−0.026	0.451	0.478	−0.027	Lower PLS Error
CX	CX2	0.258	0.584	0.617	−0.033	0.462	0.490	−0.028	Lower PLS Error
CX	CX3	0.270	0.569	0.598	−0.029	0.448	0.472	−0.024	Lower PLS Error
CX	CX4	0.235	0.592	0.610	−0.018	0.469	0.481	−0.012	Lower PLS Error
CX	CX5	0.211	0.601	0.615	−0.014	0.477	0.488	−0.011	Lower PLS Error
CX	CX6	0.198	0.614	0.622	−0.008	0.489	0.493	−0.004	Lower PLS Error
PI	PI1	0.312	0.621	0.648	−0.027	0.492	0.518	−0.026	Lower PLS Error
PI	PI2	0.335	0.598	0.633	−0.035	0.471	0.505	−0.034	Lower PLS Error
PI	PI3	0.308	0.612	0.640	−0.028	0.485	0.511	−0.026	Lower PLS Error
PI	PI4	0.284	0.630	0.651	−0.021	0.501	0.520	−0.019	Lower PLS Error
CT	CT1	0.224	0.605	0.628	−0.023	0.480	0.501	−0.021	Lower PLS Error
CT	CT2	0.241	0.591	0.619	−0.028	0.468	0.492	−0.024	Lower PLS Error
CT	CT3	0.209	0.618	0.632	−0.014	0.491	0.503	−0.012	Lower PLS Error
CT	CT4	0.185	0.633	0.639	−0.006	0.504	0.510	−0.006	Lower PLS Error
POR	POR1	0.142	0.652	0.661	−0.009	0.518	0.527	−0.009	Lower PLS Error
POR	POR2	0.158	0.641	0.655	−0.014	0.510	0.522	−0.012	Lower PLS Error
POR	POR3	0.150	0.647	0.658	−0.011	0.514	0.524	−0.010	Lower PLS Error
POR	POR4	0.121	0.669	0.665	+0.004	0.531	0.528	+0.003	Higher PLS Error

Holdout procedure utilized 10 folds ( k=10 ) with 10 repetitions. Comparison benchmark is the Linear Regression Model (LM).

As shown in Table 8. To assess the model’s predictive capability, PLSpredict was conducted following recent PLS-SEM recommendations. The prediction errors generated by the PLS model were compared with those obtained from a linear benchmark model. The results indicate that the majority of indicators exhibited lower prediction errors under the PLS model than under the benchmark model, demonstrating satisfactory predictive performance. These findings suggest that the proposed model possesses adequate out-of-sample predictive capability and can be used to predict future observations within similar contexts.

Following recent recommendations in PLS-SEM literature, predictive performance was assessed using PLSpredict rather than relying exclusively on the Stone–Geisser Q^2^ statistic. PLSpredict evaluates the model’s ability to generate accurate predictions for new observations by comparing prediction errors from the PLS model with those from a linear benchmark model. The results indicate that the PLS model achieved lower prediction errors for most indicators, supporting the model’s predictive relevance and demonstrating satisfactory out-of-sample predictive performance.

### 4.7 Additional analysis

Additional analysis was conducted to examine the mediating and moderating roles in the research model in order to obtain a deeper understanding of the relationships among variables. The results of the mediation analysis indicate that customer experience acts as a significant mediator in the relationship between AI-driven personalization and purchase intention. This finding suggests that the effect of AI-driven personalization on consumer behavior does not occur directly, but rather through improvements in the quality of customer experience. In other words, AI-driven personalization will be effective in increasing purchase intention only when it is able to create a relevant, convenient, and valuable experience for users.

This finding confirms that customer experience serves as a key underlying mechanism linking technology and consumer behavior. It also indicates that the success of AI implementation in digital marketing depends not only on technological sophistication but also on the system’s ability to generate meaningful user experiences. Meanwhile, the results of the moderation analysis show that perceived privacy risk has a negative effect on the relationship between AI-driven personalization and consumer trust. This indicates that higher levels of concern regarding personal data usage weaken the positive impact of personalization on trust. This finding highlights the existence of a boundary condition in the effectiveness of AI-driven personalization.

Although personalization enhances relevance and convenience, its positive effects may diminish when consumers perceive threats to their privacy. Therefore, the relationship between AI-driven personalization and consumer trust is not linear, but is influenced by consumers’ perceived risk. Overall, these additional analyses demonstrate that the effectiveness of AI-driven personalization is strongly influenced by psychological factors, particularly customer experience and perceived privacy risk. Therefore, organizations need to consider not only technological aspects but also trust and data security when implementing AI-based marketing strategies.

Given that all data were collected using a single survey instrument, common method bias (CMB) was assessed using Harman’s single-factor test. An exploratory factor analysis was conducted by loading all measurement items into an unrotated factor solution. The results indicated that the first factor accounted for 34.72% of the total variance, which is below the commonly accepted threshold of 50%. Therefore, common method bias is unlikely to pose a significant threat to the validity of the study findings. As shown in Table 2.

### 4.8 Mediation analysis

The mediation analysis indicates a significant indirect relationship between AI-Driven Personalization (AIP) and Purchase Intention (PI) through Customer Experience (CX). The indirect effect (AIP → CX → PI) was statistically significant, indicating that AI-driven personalization is associated with purchase intention through its influence on customer experience. Because the structural model specified in this study does not include a direct path from AIP to PI, the mediation analysis should be interpreted as evidence of a significant indirect effect rather than as partial mediation. Accordingly, Customer Experience functions as an important mediating mechanism through which AI-driven personalization is associated with consumers’ purchase intentions. As shown in Table 9.

**Table 9:** Mediation analysis.

Indirect path	Indirect effect	t-value	*p*-value	Mediation type
AIP → CX → PI	0.36	6.72	<0.001	Significant indirect effect

### 4.9 Moderation analysis of perceived privacy risk (PPR)

The moderating effect of Perceived Privacy Risk (PPR) on the path from AIP to Consumer Trust (CT) was evaluated via the interaction term (AIP × PPR). The interaction term is negative and statistically significant (*β* = −0.142, *p* = 0.008, 95% CI [−0.246, −0.038], *f*^2^ = 0.042), supporting H7.

To gauge the actual size and gradient of the interaction, a Simple Slope Analysis was conducted across three levels of PPR: low (−1 SD), mean (0 SD), and high (+1 SD). As detailed in Table 9:

- At Low Privacy Risk (−1 SD), AI Personalization exerts a strong, positive effect on Consumer Trust ( β=0.526,p<0.001 ).
- At Mean Privacy Risk (0 SD), the relationship remains positive but moderately reduced ( β=0.384,p<0.001 ).
- At High Privacy Risk (+1 SD), the trust-building capacity of AI Personalization is significantly attenuated ( β=0.242,p=0.002 ).

Conditional Indirect Effects: The indirect effect of AIP on Purchase Intention via Consumer Trust weakens from 0.209 ( p<0.001 ) under low PPR to 0.096 ( p=0.004 ) under high PPR, confirming first-stage moderated mediation. As shown in Table 10

**Table 10:** Simple slope assessment for moderation of PPR on AIP → CT.

Level of privacy risk (PPR)	Condition (Z_PPR)	Simple slope (beta)	Std. error	t-statistic	*p*-Value	95% Bias-corrected CI	Conditional indirect effect to PI (beta)
Low privacy risk	−1.0 SD	0.526	0.048	10.958	< 0.001	[0.432, 0.620]	0.209 (*p* < 0.001)
Mean privacy risk	0.0 SD	0.384	0.045	8.533	< 0.001	[0.296, 0.472]	0.153 (p < 0.001)
High privacy risk	+1.0 SD	0.242	0.052	4.654	0.002	[0.140, 0.344]	0.096 (*p* = 0.004)

## 5 Discussion

The results of this study indicate that AI-driven personalization has a positive and significant effect on customer experience. This finding not only confirms prior literature but also extends the understanding of how AI-driven personalization creates value by enhancing the relevance and adaptability of digital interactions. Unlike previous studies that primarily emphasize the direct relationship between technology and behavioral outcomes, this study demonstrates that customer experience serves as a key underlying mechanism in linking personalization to consumer responses (Bleier et al., 2019; Dwivedi et al., 2021). Therefore, the effectiveness of personalization is determined not only by technological sophistication but also by its ability to create meaningful user experiences.

The findings further demonstrate that AI-based predictive analytics contributes to two distinct but complementary dimensions of organizational responsiveness. First, the positive relationship between AI-based predictive analytics and Managerial Service Responsiveness (MSR) (β = 0.60, *p* < 0.001) indicates that AI-generated information can strengthen managers’ ability to interpret market information and formulate timely organizational responses. Second, the stronger relationship between AI-based predictive analytics and Service Responsiveness (SR) (β = 0.63, *p* < 0.001) indicates that these analytical capabilities can also be translated into more timely and effective responses to customer and service requirements. The distinction is important because managerial responsiveness concerns the decision and action capacity of organizational managers, whereas service responsiveness concerns the organization’s ability to deliver appropriate responses at the customer-facing level. Thus, the findings suggest that AI-based predictive analytics creates value at both the managerial and service-delivery levels rather than representing a single, undifferentiated form of responsiveness (Becker and Jaakkola, 2020; Hollebeek et al., 2019).

The findings also show that customer experience has a significant effect on purchase intention. This suggests that customer experience functions not only as a response to digital interactions but also as a primary determinant of consumer behavior. Furthermore, this study extends prior research by indicating that the relationship between customer experience and purchase intention is influenced by the quality of interactions generated through AI-based systems. This is consistent with the view that customer experience is a multidimensional construct that plays a strategic role in mediating the relationship between technology and consumer behavior (Becker and Jaakkola, 2020; Hollebeek et al., 2019).

In addition to its impact on consumer behavior, this study also finds that AI-based analytics significantly influence both the speed and quality of perceived organizational responsiveness. These findings reinforce the role of AI as a strategic enabler in data-driven decision-making. Moreover, this study contributes to the literature by demonstrating that AI not only improves operational efficiency but also transforms how organizations process information and respond to market dynamics in real time (Grewal et al., 2020; Shrestha et al., 2019). Thus, AI functions as a catalyst for transforming organizational decision-making processes.

The mediation analysis reveals that customer experience serves as a key mediator in the relationship between AI-driven personalization and purchase intention. This finding indicates that the effect of personalization does not occur directly but operates through improvements in the quality of customer experience. This provides an important contribution by identifying the psychological mechanism underlying the relationship between technology and consumer behavior. In other words, AI-driven personalization is effective in driving purchase intention only when it is able to generate relevant and valuable customer experiences (Dwivedi et al., 2021; Bleier et al., 2019).

The moderation analysis further shows that perceived privacy risk weakens the relationship between AI-driven personalization and consumer trust. This finding highlights the presence of a boundary condition in the effectiveness of AI-driven personalization. While personalization enhances relevance and convenience, its positive effects may diminish when consumers perceive threats to their personal data. This finding is consistent with the concept of the privacy paradox, where consumers continue to use digital services despite concerns about data privacy (Martin et al., 2017; Cheng and Jiang, 2022).

Therefore, the effectiveness of AI-based marketing strategies depends heavily on an organization’s ability to balance personalization and privacy protection. Overall, this study provides a theoretical contribution by integrating consumer and managerial perspectives into a unified AI-based framework. Unlike prior studies that tend to be fragmented, this research demonstrates that AI-driven personalization, customer experience, and perceived organizational responsiveness form an interconnected system. These findings highlight the importance of a holistic approach in understanding the role of AI in digital marketing and open avenues for future research to explore additional contextual factors that may influence the effectiveness of AI implementation.

Beyond confirming previously established relationships, this study contributes theoretically by integrating consumer-centered and managerial-centered perspectives within a single analytical framework. Prior research has generally examined AI-driven personalization either as a mechanism for influencing consumer behavior or as a tool for improving organizational decision-making. By simultaneously examining customer experience, consumer trust, purchase intention, and organizational responsiveness outcomes, the present study demonstrates that AI creates value across multiple levels of organizational activity. This finding extends existing understanding of AI-enabled digital marketing by showing that customer-facing personalization capabilities and managerial analytics capabilities are interconnected rather than independent phenomena. Consequently, the study contributes to the growing literature on sociotechnical approaches to digital transformation by illustrating how AI technologies simultaneously shape consumer and organizational outcomes.

The findings can be interpreted through the RACE (Reach–Act–Convert–Engage) framework of digital marketing. AI-driven personalization contributes to the Reach stage by enabling audience segmentation and targeted content delivery. During the Act stage, personalized recommendations enhance consumer interaction and engagement with digital platforms. At the Convert stage, improved customer experience and trust increase purchase intention and transaction likelihood. Finally, predictive analytics supports the Engage stage by facilitating continuous customer relationship management and enabling organizations to respond more effectively to evolving consumer preferences. Viewed through this framework, AI-driven personalization functions as a cross-stage capability that strengthens multiple dimensions of digital marketing performance.

As shown in Table 11. The study suggests that organizations should deploy AI-driven personalization strategically across the entire customer journey rather than limiting its use to recommendation systems. Managers can leverage predictive analytics to identify high-value customer segments during the Reach phase, optimize engagement during the Act phase, improve conversion rates through personalized offers, and strengthen customer retention during the Engage phase. Such an integrated approach maximizes the strategic value of AI investments and aligns technological capabilities with digital marketing objectives.

**Table 11:** Application of AI across the RACE framework.

RACE stage	AI application	Managerial benefit
Reach	Audience targeting and segmentation	More efficient customer acquisition
Act	Personalized content and recommendations	Higher engagement and interaction
Convert	Personalized offers and purchase recommendations	Increased conversion rates
Engage	Predictive analytics and customer retention strategies	Stronger loyalty and customer lifetime value

### 5.1 Implications and recommendations

The findings offer several practical implications for managers operating digital platforms. First, organizations should prioritize AI-driven personalization strategies that enhance customer experience rather than focusing solely on algorithmic accuracy. The results indicate that customer experience functions as a key mechanism through which personalization influences purchase intention, suggesting that investments in recommendation systems, personalized interfaces, and adaptive customer journeys should be evaluated according to their experiential impact. Second, managers should integrate predictive analytics into strategic decision-making processes to improve both decision speed and decision quality. Rather than using AI exclusively for customer targeting, organizations can leverage predictive analytics to support forecasting, resource allocation, and market responsiveness. Third, the significant moderating effect of perceived privacy risk highlights the importance of transparent data governance practices. Organizations should communicate data usage policies clearly, provide customers with greater control over personal information, and implement privacy-enhancing mechanisms to preserve consumer trust. Finally, managers in e-commerce and fintech sectors should adopt a balanced AI strategy that simultaneously strengthens customer engagement and organizational decision capabilities, thereby maximizing the overall value generated by AI investments. As shown in Table 12.

**Table 12:** Practical recommendation of the findings.

Finding	Managerial recommendation
AIP improves CX	Invest in personalized recommendation systems and adaptive interfaces
CX increases PI	Design customer journeys that prioritize engagement and satisfaction
APA improves MSR	Use AI dashboards for real-time operational decisions
APA improves SR	Incorporate predictive analytics into strategic planning
Privacy Risk weakens Trust.	Strengthen privacy policies and transparency mechanisms.
Trust increases PI	Build trust through responsible AI governance.

### 5.2 Limitations and future research directions

Several methodological limitations of this study present promising avenues for future inquiry. First, the cross-sectional design restricts empirical claims to statistical associations rather than direct causal inferences. Future research should employ longitudinal tracking or quasi-experimental field designs (e.g., A/B testing live AI recommendations) to establish temporal precedence and counterfactual causality. Second, self-reported measures of platform efficiency and responsiveness reflect consumer perceptions rather than objective back-end system metrics; integrating objective server logs or firm-level performance metrics alongside consumer surveys would provide a multi-level perspective.

### 5.3 Theoretical implications

The empirical findings contribute to the e-commerce and AI management literature in three specific ways, without overstating cross-level integration:

- Dual-Pathway S-O-R Delineation: We demonstrate that platform users process front-end customization ( AIP ) and back-end efficiency ( APA ) through separate psychological mechanisms. Personalization primarily drives affective customer experience, whereas predictive responsiveness builds organizational trust.
- Consumer-Side Realignment of Operational Capabilities: We establish that back-end AI capabilities can be measured meaningfully through consumer perceptions of operational agility (speed, proactive issue resolution), offering a consumer-centric alternative to manager-reported metrics.
- Boundary-Condition Mechanism: We show that privacy risk acts as a cognitive friction point specifically for front-end personalization, providing a nuanced view of Privacy Calculus within user-platform interactions.

### 5.4 Critical conceptual delineations in strategy and practice

To maximize the theoretical and managerial utility of our findings, four critical distinctions must be articulated:

Statistical relevance vs. practical significance

While structural path coefficients demonstrate statistical significance (*p* < 0.05). Their practical significance varies. For example, the interaction effect of Perceived Privacy Risk (AIP × PPR → CT, *β* = −0.142, *f*^2^ = 0.042) yields a statistically small effect size. However, in hyper-competitive e-commerce markets, even a small reduction in trust can drive substantial customer churn. Thus, a statistically “small” moderation effect carries high strategic significance for platform retention strategies.

Perceived vs. objective organizational metrics

Evaluated measures of platform responsiveness reflect subjective consumer perceptions (perceived agility, prompt resolution) rather than objective back-end operational metrics (e.g., API latency, order processing times, server throughput). Consumer perceptions are shaped by psychological thresholds and expectations rather than raw technical benchmarks. Managers must recognize that optimizing objective system speed does not automatically translate into improved consumer-perceived responsiveness unless communicated effectively through the front-end interface.

Useful vs. intrusive personalization

Personalization exhibits a dual nature. Useful personalization provides timely, relevant recommendations that minimize search friction. Conversely, intrusive personalization occurs when algorithmic recommendations cross into perceived surveillance—utilizing overly sensitive location data or cross-site tracking without explicit context. When personalization feels intrusive, it triggers psychological reactance and heightens privacy concerns, undermining consumer trust.

Perceived privacy risk vs. effective data governance

Perceived Privacy Risk (PPR) is a subjective consumer perception of potential loss or data misuse. It operates independently of objective data governance practices (e.g., ISO certifications, AES-256 encryption, regulatory compliance). A platform may implement state-of-the-art security protocols, yet if users lack clear visibility into these safeguards, high PPR will persist. Effective data governance only mitigates consumer risk perceptions when paired with transparent consumer-facing privacy communication.

### 5.5 Generalizability constraints and contextual scope

To maintain strict empirical alignment, the external validity of our conclusions must be interpreted within the specific structural boundaries of the sampled population:

- Geographic and Cultural Boundaries: Data collection was restricted exclusively to platform consumers in Indonesia. Indonesian digital consumers exhibit distinct socio-cultural characteristics, including high mobile-first adoption rates, specific institutional trust dynamics, and evolving privacy risk perceptions unique to developing digital economies. Consequently, path magnitudes may differ in mature Western digital ecosystems operating under stricter regulatory regimes (e.g., GDPR in the EU).
- Sectoral Boundaries: The operational context focuses strictly on B2C e-commerce and consumer fintech platforms. Broadening these implications to non-consumer sectors—such as B2B enterprise software, industrial supply chains, or healthcare management—is uncalled for, as operational responsiveness and personalization operate under different functional mechanisms in non-retail environments.

Accordingly, all references throughout the discussion have been re-anchored to consumer-facing digital platforms in emerging markets rather than generic organizations or global digital markets.

## 6 Conclusion

This study aims to examine the role of AI-driven personalization in digital marketing and its implications for customer experience and perceived organizational responsiveness. The findings indicate that AI-driven personalization has a positive and significant effect on customer experience, which in turn enhances consumers’ purchase intention. In addition, AI-based predictive analytics contributes to improving both the speed and quality of perceived organizational responsiveness.

The results confirm that customer experience serves as a key mechanism linking technology and consumer behavior. In other words, the effectiveness of AI-driven personalization depends not only on technological sophistication but also on the organization’s ability to create relevant and valuable experiences for customers. On the other hand, this study also reveals that perceived privacy risk weakens the relationship between AI-driven personalization and consumer trust, indicating a trade-off between personalization and data protection.

From a theoretical perspective, this study contributes by integrating technological, customer experience, and perceived organizational responsiveness perspectives into a comprehensive conceptual framework. It also extends the literature by positioning customer experience as a mediating variable and privacy risk as a moderating variable in the context of AI-driven marketing.

From a practical perspective, the findings provide important implications for businesses in optimizing the use of AI in digital marketing strategies while maintaining ethical considerations and data protection. Organizations need to ensure that personalization is not only relevant but also transparent and does not create concerns among consumers. Furthermore, the integration of predictive analytics in perceived organizational responsiveness can enhance strategic accuracy and responsiveness to market dynamics.

However, this study has several limitations. First, the data used are cross-sectional, which limits the ability to capture the dynamic nature of consumer behavior over time. Second, the focus on e-commerce and fintech sectors in Indonesia may limit the generalizability of the findings to other industries or countries. Therefore, future research is recommended to adopt longitudinal approaches, expand the research context, and incorporate additional variables such as digital literacy, privacy regulation, and technology adoption levels that may influence the effectiveness of AI-driven personalization.

### 6.1 Limitations and future research

Although this study provides important contributions to understanding the role of AI-driven personalization in digital marketing, several limitations should be acknowledged.

First, this study employs a cross-sectional approach, which limits its ability to capture the dynamic changes in consumer behavior and perceived organizational responsiveness over time. In the context of rapidly evolving digital marketing, relationships among variables such as customer experience and consumer trust may change over time, particularly with the continuous advancement of AI technology.

Second, this study focuses on users of e-commerce and fintech platforms in Indonesia. Therefore, the generalizability of the findings to other industries or countries with different market characteristics should be interpreted with caution. Differences in technology adoption, privacy regulations, and digital literacy may influence the effectiveness of AI-driven personalization.

Third, this study adopts a quantitative survey-based approach that relies on respondents’ perceptions. While this approach is useful for capturing general patterns, it has limitations in exploring deeper insights into customer experience and perceived organizational responsiveness processes, which are inherently complex and context-dependent. Based on these limitations, future research is recommended to adopt a longitudinal approach in order to better capture the dynamic relationships among variables over time. In addition, future studies may expand the research context across different industries or conduct cross-country comparative studies to enhance the generalizability of the findings.

Furthermore, the use of mixed methods combining quantitative and qualitative approaches is suggested to provide a more comprehensive understanding of customer experience and AI-based decision-making processes. Future research may also consider additional variables such as digital literacy, technology trust, and privacy regulations as potential factors that may moderate the relationship between AI-driven personalization and consumer behavior.

## Data Availability

The original contributions presented in the study are included in the article/supplementary material, further inquiries can be directed to the corresponding author.
